# Fungal Endophytes Control *Fusarium graminearum* and Reduce Trichothecenes and Zearalenone in Maize

**DOI:** 10.3390/toxins10120493

**Published:** 2018-11-24

**Authors:** Mohamed F. Abdallah, Marthe De Boevre, Sofie Landschoot, Sarah De Saeger, Geert Haesaert, Kris Audenaert

**Affiliations:** 1 Centre of Excellence in Mycotoxicology and Public Health, Department of Bioanalysis, Faculty of Pharmaceutical Sciences, Ghent University, 9000 Ghent, Belgium; Marthe.DeBoevre@UGent.be (M.D.B.); Sarah.DeSaeger@UGent.be (S.D.S.); 2 Laboratory of Applied Mycology and Phenomics, Department of Plants and Crops, Faculty Bioscience Engineering, Ghent University, 9000 Ghent, Belgium; Sofie.Landschoot@UGent.be (S.L.); Geert.Haesaert@UGent.be (G.H.)

**Keywords:** *Fusarium graminearum*, biocontrol, mycotoxins, endophytes, biogenic volatiles, deoxynivalenol, zearalenone, biological control, maize

## Abstract

*Fusarium graminearum* can cause Giberella Ear Rot (GER) and seedling blight in maize, resulting in major yield losses. Besides GER, the infected grains are consequently contaminated with multiple mycotoxins of *F. graminearum*. Zearalenone and trichothecenes, such as deoxynivalenol and its acetylated forms, are among the major mycotoxins associated with *F. graminearum* infection in maize. In the current work, we explored the effect of the endophytic fungal genera of *Epicoccum* and *Sordaria*, to control *F. graminearum* infection in comparative trials with *Piriformospora* spp., an elusive endophytic genus. Furthermore, we investigated the effect of these endophytes on zearalenone, deoxynivalenol, and 15-acetyldeoxynivalenol levels using in vitro and in planta assays. As plants are endowed with several detoxification mechanisms comprising e.g., glucosylation of trichothecenes, the effect of the isolated fungal endophytes on the deoxynivalenol-3-glucoside level was also assessed. In general, results showed a considerable variability in the antifungal activity, both among species and among isolates within one species. Additionally, the effect on mycotoxin levels was variable, and not necessarily related to the antifungal activity except for zearalenone levels which were consistently reduced by the endophytes. These results highlight the great potential of certain endophytic fungal strains as new biocontrol agents in agricultural science.

## 1. Introduction

Cereals are a major daily source of calories for humans. Securing enough food for the continuously expanding population—especially in the less developed countries—necessitates an increase in food production by 50% to 70% over the next 30 years [1]. Despite being a global staple crop, maize (*Zea mays* L.) is a susceptible host for a wide range of plant pathogens including mycotoxigenic fungi e.g., *Fusarium graminearum* [Schwabe] which is one of the predominant members involved in maize Giberella Ear Rot (GER), stalk rot, and seedling blight in maize [2,3]. Beside the significant economic losses due to the significant decrease in yield quantity and quality, the interest in the fungus is particularly fed by its production of several mycotoxins. These mycotoxins include zearalenone (ZEN) and several trichothecenes which comprise a large family of compounds, e.g., deoxynivalenol (DON), 15-acetyldeoxynivalenol (15-ADON), 3-acetyldeoxynivalenol, and nivalenol [4]. A database for *F. graminearum* in Europe showed that the 15-ADON chemotype is the predominant one in wheat and maize in recent years with an overall distribution in cereals up to 83% [5].

DON and its acetylated forms interfere with the cellular protein synthesis, and clinical manifestations as feed refusal and vomiting are described, while ZEN as a potent non-steroidal mycoestrogen, competitively binds to estrogen receptors causing reproductive disorders in female farm animals and humans [4]. Furthermore, the simultaneous production of all these mycotoxins adds complexity due to the potential additive or synergistic outcome [6]. Moreover, modified forms of mycotoxins are an additional alerting issue, which can be reconverted to their parent metabolites upon digestion in animal and human bodies [7]. Excluding the presence of these forms such as deoxynivalenol-3-glucoside (DON3G), underestimates the contamination level of the total mycotoxins and the consequent mycotoxin exposure.

Although *F. graminearum* has always been present in agro-ecosystems, intensification of crop production combined with a narrow genepool in crops promotes spread, virulence, and diversity of this pathogen. The farmer interferes via an integrated approach comprising prevention, intervention, and remediation strategies. Consequently over the last decades, different strategies such as, (1) sound crop rotation; (2) good tillage practices; (3) seeding resistant cultivars; (4) introducing resistance gene(s) against these pathogens; (5) biological control; (6) advanced functional genomics tools to suppress expression of fungal genes responsible for colonization and mycotoxin synthesis; and (7) applying pesticides as chemical control, have been proposed for a meaningful plant disease management. However, driven by health and environmental concerns the European Union (EU) developed a new EU directive on sustainable use of pesticides (2009/128/EC). This directive aims at reducing the use and risks of pesticides by implementing a more integrated approach by November 2018. In a search for alternative methods to secure food and feed production, the industry shifted to agricultural inoculants composed of bioactive bacterial and fungal isolates obtained from natural rhizospheric soils that exhibit significant plant-growth promoting and/or biocontrol activities. Although there are a large number of published reports on biological control to provide plant protection against many diseases, this area is still in its infancy as most of the documented Biological Control Agents (BCAs) are still stuck at the in vitro lab-scale, and are generally not commercialized, some exceptions not withstanding [8]. Also, the efficacy of BCAs in the field is arguably due to their durability [9]. Thus, exploring new BCAs is required and timely. Endophytic fungi living in close association with the host might provide a valuable new type of biocontrol agent [10]. The modes of action of endophytes are broad from stimulation of the host defense system to the production of antimicrobial secondary metabolites [11,12]. Numerous publications demonstrate the growing interest in using BCAs to control *F. graminearum* infection [13,14,15]. However, reports discussing the potential biocontrol effect of BCAs against the pathogen in maize are limited [8,16,17]. 

In the present work, we started from the hypothesis that stubbles/straw residues may harbor several antagonistic fungi that are adapted to local environmental conditions, and can be used as BCAs to effectively prevent fungal infection through reduction of the pathogen inoculum. Several fungal endophytes from various crop residues and soil samples were isolated and identified. In a second part of this research, their ability to inhibit *F. graminearum* growth and the production of *F. graminearum* mycotoxins, including DON3G as an important modified mycotoxin in the plant, using in vitro (contact and volatile) and in planta (maize pot experiments) was investigated.

## 2. Results

### 2.1. Isolation and Identification of New Endophytic BCAs

Screening for new endophytes in several maize stubbles and soil samples resulted in isolation of five strains of three different species (Table 1). All the isolates were grown on Potato Dextrose Agar (PDA) media, and were identified by their macroscopic and microscope shapes of the mycelia and spores. The three isolates of *Epicoccum nigrum* produced a light yellowish brown pigment. One isolate of *Sordaria fimicola*, was identical in colony shape and color to the reference strain MUCL 29304. Using BLAST search, based on Identity Matching and Query Cover, the identification of the isolates was confirmed, and all the isolates were 100% identical to the corresponding isolates present in the BLAST database. After identification, the strains were used to evaluate their ability to effectively control *F. graminearum* growth, mycotoxin production, and infection in maize plant.

### 2.2. Effect of Endophytic BCAs on F. graminearum Growth and Mycotoxin Levels under Direct Plating Assay

**Figure 1:**
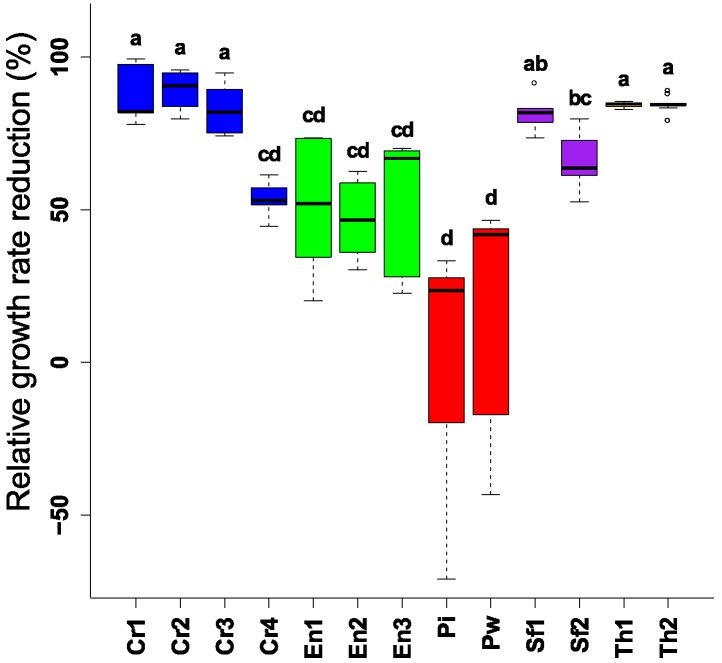
Reduction of mycelial growth of *F. graminearum* after challenging by different endophytic and non-endophytic Biological Control Agents (BCAs) in plating assay (4 days). Different letters above the boxes point to significant differences according to a Dunn test. Cr1 (*C. rosea* CBS 100502); Cr2 (*C. rosea* CBS 102.94); Cr3 (*C. rosea* CBS 100494); Cr4 (*C. rosea* UG 1116); En1 (*E. nigrum* UG 11701); En2 (*E. nigrum* UG 11702); En3 (*E. nigrum* UG 11703); Pi (*P. indica* DSM11827); Pw (*P. williamsii* DAR29830); Sf1 (*S. fimicola* UG 1016); Sf2 (*S. fimicola* MUCL 29304); Th1 (*T. harzianum* CBS 226.95); Th2 (*T. harzianum* CBS 243.71).

Challenging *F. graminearum* with different endophytic BCAs for four days showed antagonism and inhibition of the mycelial growth (Figure 1). The well-known biocontrol fungal genera (*Trichoderma* spp., and *Clonostachys* spp.) showed the highest reduction in *F. graminearum* growth (70–99% reduction). However, one isolate of *Sordaria fimicola* (*S. fimicola* UG 1016) had a similar effect compared to the reference strains. All other strains, except the *Piriformospora* strains, could restrain *F. graminearum* growth, but they were less effective than the reference strains. No difference in the biocontrol performance between the strains of the fungal endophytic species was observed.

**Figure 2:**
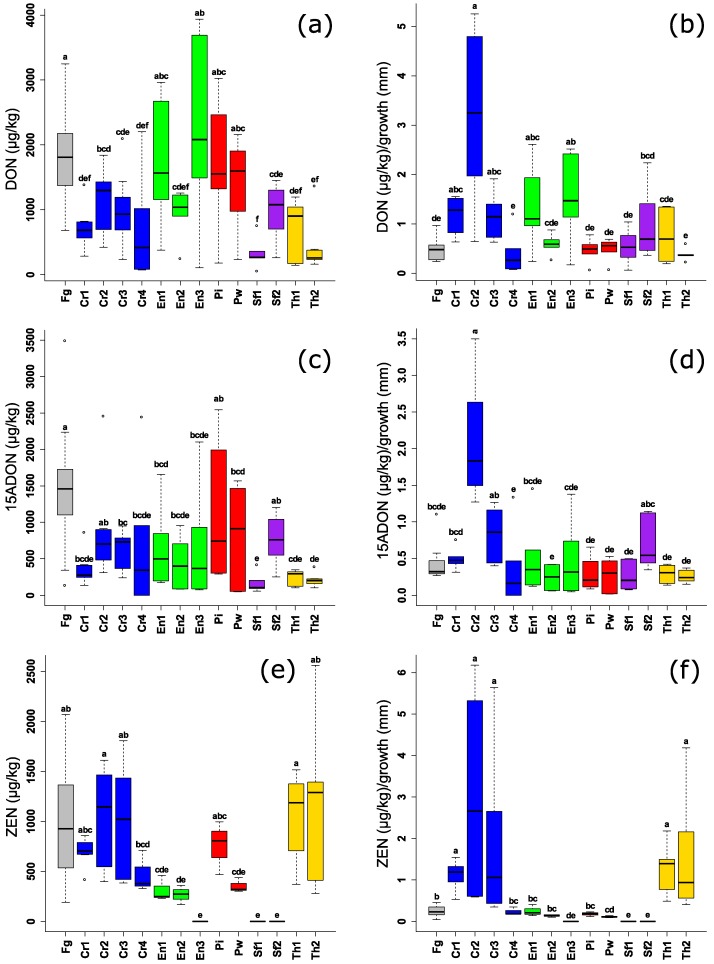
(**a**,**c**,**e**) Levels of *F. graminearum* mycotoxins (µg/kg) after challenging by different endophytic and non-endophytic BCAs in plating assay (4 days). (**b**,**d**,**f**) Levels of *F. graminearum* mycotoxins (µg/kg) in relation to mycelial growth (mm) of *F. graminearum*. Different letters above the boxes point to significant differences according to a Dunn test. Fg (*F. graminearum* PH1); Cr1 (*C. rosea* CBS 100502); Cr2 (*C. rosea* CBS 102.94); Cr3 (*C. rosea* CBS 100494); Cr4 (*C. rosea* UG 1116); En1 (*E. nigrum* UG 11701); En2 (*E. nigrum* UG 11702); En3 (*E. nigrum* UG 11703); Pi (*P. indica* DSM11827); Pw (*P. williamsii* DAR29830); Sf1 (*S. fimicola* UG 1016); Sf2 (*S. fimicola* MUCL 29304); Th1 (*T. harzianum* CBS 226.95); Th2 (*T. harzianum* CBS 243.71).

By measuring the absolute mycotoxin levels in the same PDA plates, the effect of BCAs was variable, both among species and among the different isolates of the same species, especially for DON and 15-ADON (Figure 2a,c). Both isolates of the endophytic *S. fimicola* (UG 1016 and MUCL 29304) and one isolate of the endophytic *E. nigrum* spp., (UG 11702) could decrease DON levels to the same extent, or even better, than the reference BCAs. *C. rosea* UG 1116 performed on average equally well as the other reference strains of *Clonostachys* spp., but the results were inconsistent (high variability between the replicates). Levels of 15-ADON were significantly reduced in case of *S. fimicola* UG 1016, while other endophytic BCAs could also reduce the mycotoxin levels but with a high variation. Three endophytes (*S. fimicola* UG 1016 and MUCL 29304 and *E. nigrum* UG 1703) resulted in a complete reduction of ZEN. Also, *C. rosea* UG 1116, *E. nigrum* (UG 11701 and UG 11702) and *P. williamsii* DAR29830 were consistent in their effect against ZEN production. Surprisingly, all the reference strains had no effect on ZEN levels i.e., no significant difference from the positive control (*F. graminearum* PH1).

By investigating mycotoxin levels relative to the mycelial growth area, we observed that none of the endophytes realized an active reduction of DON and 15-ADON (Figure 2b,d). Therefore, the reduction of mycotoxin levels is solely related to suppression of fungal growth. Remarkably, one reference strain, *C. rosea* CBS 102.94, stimulated the production of both mycotoxins.

Interestingly, *S. fimicola* (UG 1016 and MUCL 29304), *E. nigrum* UG 1703, and *P. williamsii* DAR29830 resulted in a consistent reduction of ZEN levels both in absolute values, and relative to fungal growth (Figure 2e,f) suggesting a dual inhibition effect, directly through inhibition of the ZEN synthesis and indirectly through inhibition of fungal growth while the well-known biocontrol genera, *Trichoderma* spp. and *Clonostachys* spp., were not effective in reduction of ZEN. Both isolates of *Piriformospora* spp. were able to suppress ZEN production without a reduction of fungal growth (Figure 1), however, the difference between *P. indica* DSM11827 and the control was not significant.

### 2.3. Effect of Endophytic BCAs on F. graminearum Growth and Mycotoxin Levels in the Volatile Assay

**Figure 3:**
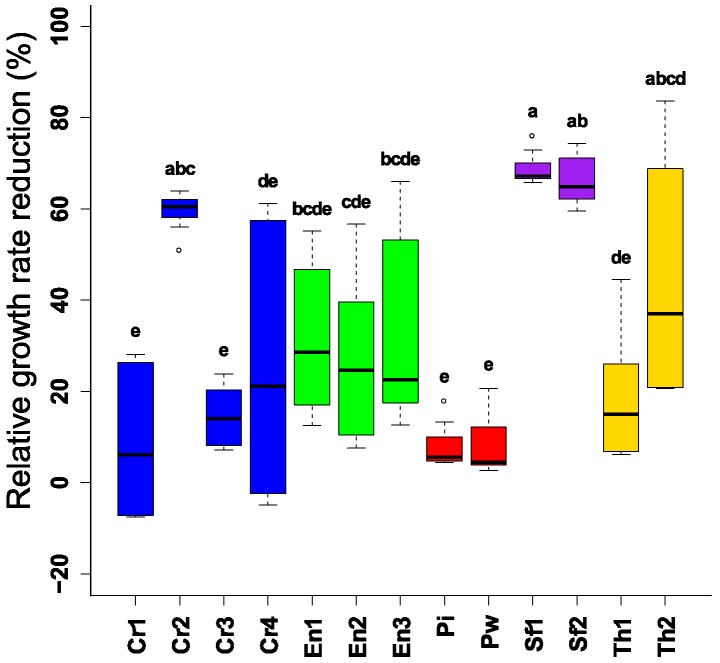
Reduction of mycelial growth of *F. graminearum* after challenging by different endophytic and non-endophytic BCAs in volatile assay (3 days). Different letters above the boxes point to significant differences according to a Dunn test. Fg (*F. graminearum* PH1); Cr1 (*C. rosea* CBS 100502); Cr2 (*C. rosea* CBS 102.94); Cr3 (*C. rosea* CBS 100494); Cr4 (*C. rosea* UG 1116); En1 (*E. nigrum* UG 11701); En2 (*E. nigrum* UG 11702); En3 (*E. nigrum* UG 11703); Pi (*P. indica* DSM11827); Pw (*P. williamsii* DAR29830); Sf1 (*S. fimicola* UG 1016); Sf2 (*S. fimicola* MUCL 29304); Th1 (*T. harzianum* CBS 226.95); Th2 (*T. harzianum* CBS 243.71).

As biogenic volatile compounds might provide new opportunities to control plant pathogens, we explored the capacity of these volatile compounds to reduce *F. graminearum* and the subsequent mycotoxin production. Both isolates of the endophyte *Sordaria fimicola* spp. (UG 1016 and MUCL 29304) suppressed the mycelial growth by 60% to 70%. Other tested endophytic strains had an inhibitory effect, however this was, on average, limited (<50%) (Figure 3). Furthermore, there was a clear effect of the volatiles on color and pigment production by *F. graminearum* (data not shown). Similar to the direct plating assay, no difference in the biocontrol capacity between the strains of the fungal endophytic species was observed, and *Piriformospora* spp. showed the lowest antifungal effect.

**Figure 4:**
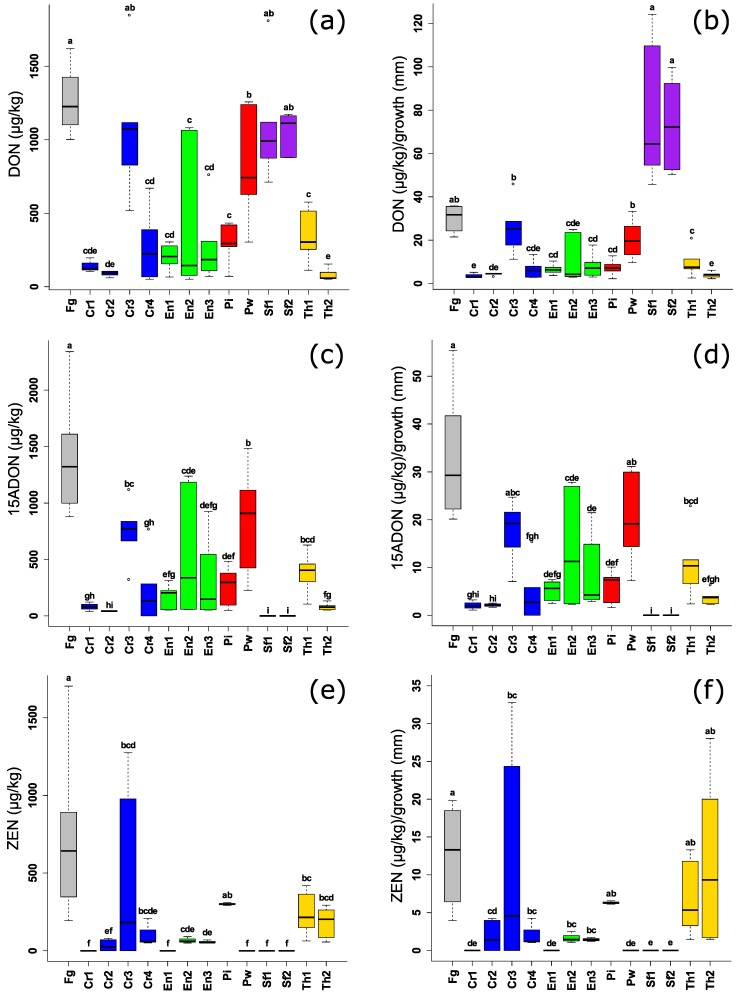
(**a**,**c**,**e**) Levels of *F. graminearum* mycotoxins (µg/kg) after challenging *F. graminearum* by different endophytic and non-endophytic BCAs in volatile assay (3 days). (**b**,**d**,**f**) Levels of *F. graminearum* mycotoxins (µg/kg) in relation to mycelial growth (mm) of *F. graminearum*. Different letters above the boxes point to significant differences according to a Dunn test. Fg (*F. graminearum* PH1); Cr1 (*C. rosea* CBS 100502); Cr2 (*C. rosea* CBS 102.94); Cr3 (*C. rosea* CBS 100494); Cr4 (*C. rosea* UG 1116); En1 (*E. nigrum* UG 11701); En2 (*E. nigrum* UG 11702); En3 (*E. nigrum* UG 11703); Pi (*P. indica* DSM11827); Pw (*P. williamsii* DAR29830); Sf1 (*S. fimicola* UG 1016); Sf2 (*S. fimicola* MUCL 29304); Th1 (*T. harzianum* CBS 226.95); Th2 (*T. harzianum* CBS 243.71).

For mycotoxins, most of the endophytic BCAs significantly suppressed DON, 15-ADON and ZEN in comparison with the positive control (Figure 4a,c,e) although, high variation was observed between the species especially for DON and 15-ADON. DON inhibition was highest in the case of the well-known biocontrol genera of *C. rosea* and *T. harzianum*. Interestingly, the endophytic *S. fimicola* UG 1016 and MUCL 29304 had no effect against DON, but they were the best performing strains against 15-ADON and ZEN due to their complete suppression of both mycotoxins (Figure 4a,c,e). Two strains of *E. nigrum* spp. (UG 11701 and UG 11702), showed a consistent reduction for DON, 15-ADON, and ZEN which was significantly lower than the control. The two species of the endophyte *Piriformospora* spp., had a different effect on the three mycotoxins. *P. indica DSM11827* performed better in reducing DON and 15-ADON levels, while lower levels of ZEN were detected with *P. williamsii* DAR29830.

By considering mycotoxin levels relative to fungal or mycelial growth, it can be clearly seen that the tested endophytes have a consistent reduction of ZEN levels which might be related to a direct inhibition of ZEN synthesis (Figure 4f). This spectacular effect is less in the case of DON and 15-ADON due to the variation. *Sordaria fimicola* strains, however, completely suppressed 15ADON: an increase of DON levels was observed even in higher levels than the positive control (Figure 4b,d). Thus, this phenomenon indicates that the low toxin contents can be the result of the low amount of fungal biomass, and is not directly related to a reduction in the mycotoxin synthesis.

### 2.4. Effect of Endophytic BCAs on F. graminearum Growth and Mycotoxin Levels in an in planta Assay

**Figure 5:**
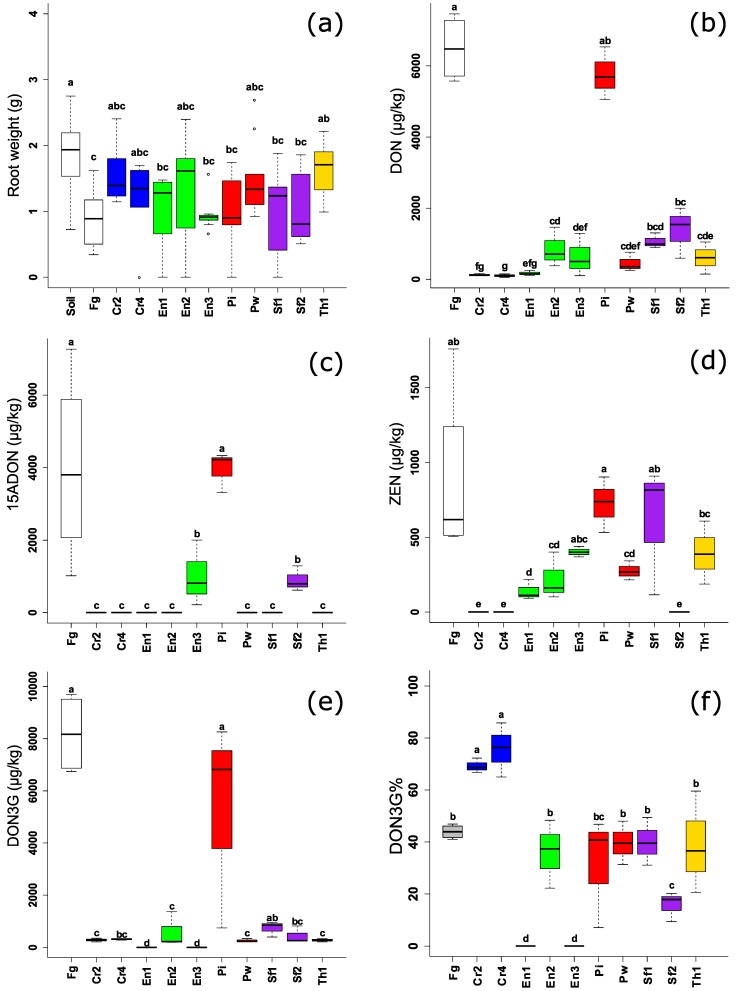
(**a**) Root weight of maize (2 weeks) challenged with *F. graminearum* and endophytic and non-endophytic BCAs. (**b**–**e**) Levels of *Fusarium* mycotoxins (µg/kg) in maize plant (2 weeks grown in greenhouse experiments) challenged with *F. graminearum* and endophytic and non-endophytic BCAs. (**f**) Percentage of DON3G levels over the total DON, 15-ADON, and DON3G in maize (2 weeks grown in greenhouse experiments). Different letters above the boxes point to significant differences according to a Dunn test. Fg (*F. graminearum* PH1); Cr2 (*C. rosea* CBS 102.94); Cr4 (*C. rosea* UG 1116); En1 (*E. nigrum* UG 11701); En2 (*E. nigrum* UG 11702); En3 (*E. nigrum* UG 11703); Pi (*P. indica* DSM11827); Pw (*P. williamsii* DAR29830); Sf1 (*S. fimicola* UG 1016); Sf2 (*S. fimicola* MUCL 29304); Th1 (*T. harzianum* CBS 226.95).

A subset of the screened isolates was tested in the in planta assay in order to evaluate their ability against *F. graminearum* PH1 pathogen. *C. rosea* CBS 102.94, *P. indica* DSM11827 and *T. harzianum* CBS 226.95 were used as reference strains in this assay. The biocontrol effect was present for the majority of endophytic BCAs, although the effect was minor and variable (Figure 5a).

On the other hand, the effect of the endophytic BCAs on mycotoxin production in planta was significant and more proliferated (Figure 5b–e). Although endophytes had a variable effect on the DON levels under in vitro conditions (Figure 2a and Figure 4a), all of them (except *P. indica* DSM11827) were able to significantly reduce DON levels in maize. Furthermore, no differences between the strains of the same species were observed except for the *Piriformospora* strains. *P. indica* DSM11827 did not reduce mycotoxin levels in planta, while *P. williamsii* DAR29830 was among the best BCAs in this assay. For 15-ADON, *P. indica* DSM11827 had no effect on reducing the mycotoxin level while other BCAs (the endophytes and the references) had a complete reduction of the mycotoxin except *E. nigrum* UG 11703 and *S. fimicola* MUCL 29304 had less effect, but still significantly different to the positive control. More variation in ZEN levels was observed in planta than in vitro for *Piriformaspora* spp., *E. nigrum* and *S. fimicola* strains. The effect exerted by endophytes on reducing DON3G levels was nearly the same on DON and 15-ADON i.e., *E. nigrum* UG 11703 and *S. fimicola* MUCL 29304 had less effect on decreasing the modified mycotoxin than other strains, while *P. indica* DSM11827 had no effect on DON3G levels. Calculating the percentage of DON3G over the total DON and 15-ADON showed that there was no effect exerted by the fungal endophytes on the conversion of the modified mycotoxin DON3G on DON in the plant, except for *C. rosea* (CBS 102.94 and UG 1116) where an increase in the conversion rate of DON3G was observed (Figure 5f).

## 3. Discussion

### 3.1. Isolation and Identification of Fungal Endophytes as New BCAs

*F. graminearum* is a widespread pathogen in small-grain cereals in several countries of the world. The initial step for a successful biocontrol strategy begins with screening, detection, and identification of strains that have a capacity to survive environmental conditions prevailing in a certain region. In the past, researchers often selected strains which were highly performant under laboratory conditions, while they were poor survivors in a soil in vivo environment. Therefore, we decided to focus on endophytic genera and species which are known to live in close association with the plant, as this trait might result in higher survival rate and activity of the BCAs compared to the classical BCAs. Beneficial endophytes are a highly diverse group of microorganisms able to colonize the plant tissue without causing obvious lesions [10]. Implementation of endophytes showed a promising tool for a worthwhile control of many plant-pathogens including *F. graminearum* in several crops such as maize [16,17]. The beneficial impacts of endophytes on the plant communities is attributed to their ability to enhance plant fitness by conferring abiotic and biotic stress tolerance, increasing biomass, and diminishing water consumption, or decreasing fitness by altering resource allocation. The selected BCAs were all reported previously for their endophytic ability to colonize different plants [18,19], however, they were isolated from soil and maize residues except for *Piriformospora* strains which were not isolated in the current work [20].

### 3.2. Effect of Endophytic BCAs on F. graminearum under In Vitro and in Planta Conditions

In general, the reduction of *F. graminearum* growth was more obvious in the dual culture assay (up to 99%) than in the volatile assay (70–80%). This may be due to direct inhibition of the pathogen growth in addition to the competition for nutrient and space available in the plate. While in the volatile assay, the inhibition is only directed by the type and the quantity of the produced biogenic volatile substance(s).

*Epicoccum nigrum* strains were previously reported to be effective BCAs against *F. graminearum* through reduction of the sporulation under a controlled bioassay on maize stubble [17]. Recently, two isolates of *E. nigrum* have been documented for their ability to inhibit *F. graminearum* in dual culture assays and on sterilized wheat grains [21]. In the present research, a similar conclusion on growth reduction was drawn, both in vitro, dual culture and volatile, and in planta assays which confirm the potential ability of *E. nigrum* to compete against the pathogen.

*E. nigrum* has been isolated from soil samples in Flanders. Although the fungus has been identified as one of the endophytic inhabitants in wheat grains [21]. It is also reported that the fungus has a protective effect against *F. verticillioides* in sugarcane with stimulation of the plant growth [22]. In the current study, the observed antifungal effect of *E. nigrum* in vitro may be attributed to the production of several antifungal compounds. Talontis et al. (2013) characterized three poly-oxygenated polyketides named epicolactone, epiccocclides A, and epiccocclides B which showed an antimicrobial effect against several pathogenic bacteria such as *Bacillus subtilis*, *Staphylococcus aureus,* and *Escherichia coli* [23]. Perveen et al. (2017) isolated 2-methyl-3-nonyl prodiginine, bis (2-ethylhexyl) phthalate, and preaustinoid A, and found that these compounds could be used as antimicrobial agents [24]. Flavipin, a yellow crystalline powder produced by *E. nigrum* and other fungi has been claimed to be involved in the inhibitory effect due to its antifungal activity [25]. However, there is no evidence of the responsible compound for the reduction of *F. graminearum* and the subsequent mycotoxin levels.

*S. fimicola* is an endophyte isolated from soil [26] and several plants [18]. This fungus is associated, but not obligated to the dung of herbivorous animals [18]. The biocontrol effect of *S. fimicola* can be related to multiple modes of action as competition for nutrients and space due to the rapid growth of the fungus and production of certain volatile compounds that may strongly suppress the fungal growth. Studies for the biocontrol effect of *S. fimicola* have been reported before in which several isolates of *Sordaria* spp. including three isolates from *S. fimicola* were able to control soil-born plant diseases in cucumber, spinach, komatsuna, and Japanese black pine [27]. Another report documented the reduction of the pathogenicity of the take-all fungus in wheat and the promotion of wheat growth [26]. In another work, this fungus significantly reduced the growth of *Bromus tectorum* [18]. Here, our findings under greenhouse conditions confirm the potential biocontrol effect of *S. fimicola* against several pathogens including *F. graminearum* in maize.

The cultivable root endophyte, *P. indica*, has been found to be involved in promoting the growth of various cereal crops such as wheat and barley [28,29], and to control the *F. graminearum* infection [30,31]. The ability of *P. indica* to promote the growth in maize has been documented [32], however, the endophyte had not been tested to control the GER in maize. Our findings were in contrast to the previously reported studies in promoting the growth rate and as a potential BCA against *F. graminearum*. One of the reasons could be related to the ability of the strain to colonize the maize and exert its protecting role, or the stimulation of the plant defense mechanism by *P. indica* was not enough to reduce the symptoms or infection. However the direct plating assay and volatile assays showed that *P. indica* is the lowest performing BCA. The same performance for *P. williamsii*, a distinguished species from *P. indica* of the genus *Piriformospora* [33] was observed in the three assays. Despite both strains of *Piriformospora* spp. having been cultivated first before the pathogen in the PDA plates and in the plant due to their slow growth rate in order to give them the time to grow, they could not suppress fungal growth. No signs for antibiosis or parasitism were observed which is in alignment with a previous work where no direct antagonistic activity of *P. indica* against different *Fusarium* isolates including *F. graminearum* was observed [34].

The *C. rosea* strains were most effective in inhibition of the growth rate (89–99.5%), except for *C. rosea* UG 1116 which resulted in less inhibition (53–57.2%). These results are in line with the inhibition rates obtained after challenging *F. graminearum* with *C. rosea* strain ACM94 [35]. In the volatile assay, there was no or only a minor effect on the mycelial growth of *F. graminearum*, except for *C. rosea* CBS 102.94 which resulted in a growth rate reduction of 60%.

### 3.3. Effect of Endophytic BCAs on Multi-Mycotoxin Production under In Vitro and in Planta Conditions

It is well known that significant reduction of mycotoxins is an essential part of an effective *F. graminearum* control. However, the number of papers discussing the effect of BCAs on *Fusarium* mycotoxins is low [8]. Reduction of mycotoxins can be achieved through direct inhibition of their synthesis. Another possibility for reduction of mycotoxin levels is the ability of some fungi to directly convert the parent mycotoxins into other conjugated forms through glycosylation [36] or sulfation [37], which results in metabolites of less toxicity to human.

In general, most of the isolated endophytes showed a better capacity to reduce ZEN levels than DON and its acetylated form. To our knowledge, this is the first paper to focus on the effect of BCAs on ZEN in the plant. Levels of ZEN were consistently suppressed by the isolated endophytes in both in vitro and in planta. For, all the three assays with *S. fimicola* (except for *S. fimicola* UG 1016 which gave variable results in planta), results for ZEN were consistent. This phenomenon is opposed to a previous study that showed the inhibitory effect of ZEN on *S. fimicola* at concentrations from 1 to 15 µg/mL on solid PDA medium [38]. No reports for the production of bioactive molecules are known to the best of our knowledge. 

We hypothesize that some volatiles are produced by the selected endophytes, and those may affect the fungal growth and mycotoxin production. Yet, there is a knowledge gap for the identity of these volatile substances, especially for those produced by *E. nigrum*. Additionally, there was variability in the performance between the tested strains of each species such as the variability between *S. fimicola* UG 1016 and *S. fimicola* MUCL 29304 in DON and 15ADON levels in dual cultural assay as well as between *P. indica* DSM11827 and *P. williamsii* DAR29830 in the plant assay. For *Piriformospora* spp., Rabiey and Shaw (2016) reported a 70% decrease in DON levels in winter wheat samples when *P. indica* was inoculated with *F. graminearum*, however, under our greenhouse conditions, there was no effect on DON or other toxins including DON3G in maize [31]. The present work showed that *P. williamsii* was highly effective in decreasing the mycotoxin levels to the same extent as the references strains. According to the obtained results on the biocontrol effect of *P. williamsii* against *F. graminearum*, further investigations need to be executed to clarify the mechanism used to affect toxins in the plant.

DON3G levels were suppressed similarly to DON for all the selected endophytes except for *P. indica*. This inhibition gives a clear indication for the biocontrol effect and the selected BCAs, however, endophytes stimulate the plant immune system against *F. graminearum*. Therefore, the indirect biocontrol through enhancement of the plant defense mechanism might be limited. The present work is also the first study dealing with the modified mycotoxin DON3G in the plant.

The effect of the fungal endophytes on DON3G levels was similar to their effect on DON levels. The reduction of DON might be driven by either direct reduction of the parent mycotoxin and/or due to enhancement of mycotoxin transformation i.e., enhancement of the masking or the modification of DON into DON3G by the plant. Both *C. rosea* CBS 102.94 and *C. rosea* UG 1116 significantly reduced DON and DON3G levels in the plant. The reduction of DON seems to be partially related to the conversion of DON into DON3G rather than inhibition of the actual production of DON by *F. graminearum* (by considering the percentage of DON3G over DON, 15ADON, and DON3G). On the other hand, *E. nigrum* UG 11701, *E. nigrum* UG 11703, and *S. fimicola* MUCL 29304 decreased the levels of DON and DON3G. Moreover, the percentage of DON3G compared to the total amount of DONs was lower compared to the infected control plants suggesting that the presence of the endophytes influences the plant’s detoxification process. Other BCAs had no effect on the conversion of DON into DON3G. These results suggest that endophytic fungi can both reduce levels of DON and modify the plant’s DON detoxification mechanism.

In conclusion, our work indicates that the five candidates, in addition to *P. williamsii*, are potential BCAs to combat with *F. graminearum* and its mycotoxins in the plant, even though no growth promotion effect was observed. Also, the variability in mycotoxin reduction may be related to the diversity and the complication of mycotoxin synthesis by *F. graminearum*. Therefore, it can be concluded that no single BCA is able to suppress the fungal toxins as they are considered one of the arsenal of fungi to cope against any environmental, biological or chemical factors that may restrain their infection and growth [39]. These candidates will be further used to evaluate the performances under field conditions to control the incidence of GER in maize (2017–2019). The new endophytic strains may be used as an alternative to the traditional BCAs as they showed the same efficacy, with an additional advantage of avoiding the drawback of T-2 toxin production in case of *Trichoderma* spp., since they harbor trichothecenes (*Tri*) genes [40]. In addition, gliotoxin and viridian produced by *T. harzianum*, *T. viride* and *T. virens* showed human toxicity and phytotoxic effect through decreasing the seed germination rate in cereals.

## 4. Conclusions

The present work demonstrates novel fungal strains that have an antifungal effect against *F. graminearum* and its mycotoxins under in vitro and in planta conditions. These strains showed promising results as effective BCAs against *Fusarium* caused diseases, especially GER. Furthermore, the study indicated the effect of these endophytes as an alternative to the well-known BCAs such as *Trichoderma* spp. and *Clonostachys* spp., The focus on the simultaneous production of multiple mycotoxins was especially striking in vitro and in planta, which has not been investigated before. A last highlight raised by the study is the paramount importance of relating the inhibition of fungal growth and mycotoxin production. This was conducted in order to conclude whether the mycotoxin inhibition is related to a direct suppression of its synthesis or due to a direct reduction caused by the inhibition of fungal growth. However, BCAs still have ecological parameters that should be defined before their commercialization and application to control pathogen growth and reducing toxins production. They can be effectively used as part of an integrated management program to replace the use of synthetic chemical toxic fungicides. Isolation and characterization of the natural metabolites responsible for *F. graminearum* growth suppression and/or mycotoxin production will enable the underlying inhibition mechanisms to be unravelled.

## 5. Materials and Methods

### 5.1. Reference Strains and F. graminearum

*F. graminearum* PH1, which produces DON, 15-ADON, and ZEN, was used in the current study. Several fungal strains known for their broad spectrum biocontrol activity were used as a reference. The strains are: two strains of *Trichoderma harzianum* spp. (CBS 243.71, Switzerland) and (CBS 226.95, England) and three strains of *Clonostachys rosea* spp. (CBS 100494, Australia), (CBS 100502, France), and (CBS 102.94, The Netherlands) which were obtained from Westerdijk Fungal Biodiversity Institute, (The Netherlands). Furthermore, two strains of the fungal endophyte, *Piriformospora indica* DSM11827 and *Piriformospora williamsii* (ex multinucleate rhizoctonia DAR29830) were supplied [20]. In addition, *Sordaria fimicola* (Roberge) Cesati and de Notaris (MUCL 29304, Argentina) was obtained from the MUCL/BCCM Agro-food and Environmental Fungal Collection (Belgium). The above mentioned strains (except *Piriformospora williamsii*) were used as reference strains for direct and indirect biocontrol effects. All the strains were grown on PDA agar plates under a UV lamp (12 h light/12 h darkness) for three weeks, and spore suspension of each strain was prepared in a final concentration of 1 × 10^6^ spores per mL, and stored in a mixture of PBS and glycerol (1:4, *v*/*v*) in 2 mL CryoTubes^®^ at −80 °C for further use.

### 5.2. Screening and Molecular Identification of Endophytic BCAs

For isolation of antagonistic endophytic fungi, several maize residues and soil samples were collected from a field with monoculture maize which had been cultivated in a no-tillage system for several years (Bottelare, Belgium; October, 2016). The maize residues were surface-sterilized by soaking in sodium hypochlorite (1%) for 2 min, washed with ethanol (70%) for 1 min, washed two times with distilled water, dried for 5 min, and subsequently placed on PDA (40 g/L) (Sigma Aldrich, Overijse, Belgium) plates containing 50 µg/mL ampicillin and 50 µg/mL chloramphenicol to suppress bacterial growth. The plates were incubated for five days at 25 °C, and mycelium plugs of the outgrowing colonies were transferred to 2 mL Eppendorf tubes containing 1 mL of sterile distilled water with PBS tablets (1 tablet/100 mL), pH 7.3 ± 0.2 at 25 °C. After vigorously shaking and performing serial dilution 1 × 10^3^, 100 µL were plated onto new PDA plates, and incubated again for five days at 25 °C to obtain monosporic isolates.

Soil samples were air dried for one week, and subsamples of the soil (10 g each) were added to 100 mL water and mixed thoroughly. From the final dilution, 100 µL was transferred to plates containing dichloran-diglycerol (DG18) agar (Oxoid, Aalst, Belgium) with 2.5 mg/L Malachite Green Agar (MGA 2.5) and 300 mg/L chloramphenicol. Again the outgrowing colonies were plated onto new PDA plates, and incubated again for five days at 25 °C to obtain pure cultures. The pure colonies were then transferred, cultured in 24-well plates, each well containing 1 mL potato dextrose broth (PDB) (Sigma Aldrich, Overijse, Belgium), and incubated at 25 °C for 5 days before molecular species identification.

For accurate species identification, fungal mycelium was transferred to 2 mL Eppendorf tubes and centrifuged for 15 min at 13,000 rpm at room temperature (RT) using SIGMA 3-18K Centrifuge (SciQuip, Shropshire, UK). The supernatants were discarded and the rest were subjected to vacuum freeze-drying (Christ Alpha 1-2 LD Plus, Osterode, Germany) for 18 h. Fungal genomic DNA extraction of 100 mg per isolate was performed using the cetyl-trimethyl-ammonium bromide (CTAB; Sigma-Aldrich, St. Louis, MO, USA) protocol with modifications for fungi [41]. Before performing PCR, the extracted DNA was measured, and adjusted to 10 ng/µL using Quantus™ Fluorometer (Promega, Leiden, The Netherlands).

PCR for single species detection was performed in a 25 µL reaction mixture. Screened isolates were amplified using the Internal Transcribed Spacer (ITS) target region with ITS 4 (5′-TCCTCCGCTTATTGATATGC-3′) and ITS 5 (5′-GGAAGTAAAAGTCGTAACAAGG-3′) primers. DNA amplification was conducted with an initial denaturation of 5 min at 95 °C followed by 35 cycles of 30 s denaturation at 95 °C, 30 s primer annealing at 50 °C, 30 s extension at 72 °C and a final extension of 5 min at 72 °C using the GeneAmp PCR system 97,000 PCR (Applied Biosystem, Foster City, CA, USA). Amplicons were separated on 1.5% (*w*/*v*) agarose gels, and visualized with ethidium bromide under UV light using the Molecular Imager^®^ Gel Doc™ XR+ System with Image Lab™ Software (BIO-RAD, Hercules, CA, USA). The PCR products were purified using the E.Z.N.A.^®^ Cycle-Pure Kit (VWR International, Leuven, Belgium). The sequences were dispatched to LGC Genomics (LGC group, Berlin, Germany) for sequencing. The identity of each isolate was obtained by using SnapGene viewer^®^ 3.3 software and a BLAST (www.ncbi.nlm.nih.gov/BLAST) search on the National Center for Biotechnology Information (NCBI) nucleotide database based on the best matching score was performed. The accession number for each isolate is presented in Table 1.

### 5.3. Assessment of Antifungal Activity of the Isolated Endophytes

#### 5.3.1. Direct Plating Assay

**Figure 6:**
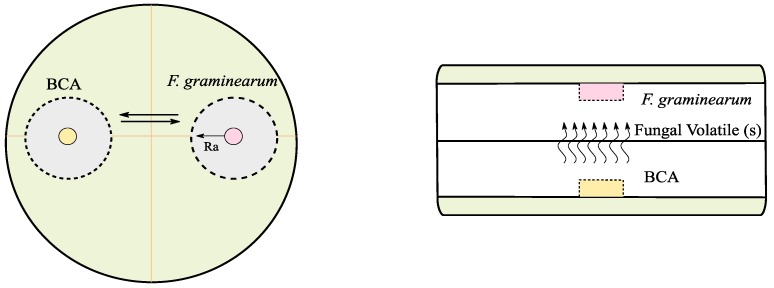
Scheme for the dual culture assay (**left**) and volatile assay (**right**). Ra, is the radius of *F. graminearum*.

The direct plating assay was performed on 90 mm petri dishes containing 25 mL PDA. Fungal discs (each 5 mm diameter) from an actively growing culture of *F. graminearum* PH1 and each antagonist were placed at a fixed distance (Figure 6, left). All the tested strains were cultured simultaneously with *F. graminearum* except *C. rosea* CBS 102.94, *P. indica* DSM11827, and *P. williamsii* DAR29830. Due to their slow growth rate, they were first cultured for four days at 25 °C, and at day five, a disc of *F. graminearum* was placed on the petri dish. The diameter of *F. graminearum* mycelium was recorded once every 24 h for four days using a digital caliber (mm). The growth rate was calculated based on these daily measurements by applying a linear regression. To obtain the percentage of *F. graminearum* growth rate reduction (in the challenged plates with BCAs), the growth rate was divided by the average of the growth rate of the *F. graminearum* control plates (without BCAs). For each isolate/*F. graminearum* PH1 combination, five replicates were done and the whole experiment was repeated three times. Also, the possible modes of action, e.g., antibiosis and/or mycoparasitism, were investigated in the same plates. In addition, the effect on pigments (quantity and color) produced by *F. graminearum* PH1 was recorded by visual observation.

#### 5.3.2. Volatile Assay

The volatile assay was designed and performed in order to evaluate the potential biocontrol effect of fungal volatile(s) produced by BCAs on *F. graminearum* growth. The assay was conducted in 45 mm petri dishes containing 10 mL PDA. A disc of 5 mm diameter from each growing BCA was placed in the center (Figure 6, right). The same was done for *F. graminearum* and the two plates were placed against each other, sealed and incubated at 25 °C for three days. All the tested strains were cultured simultaneously with *F. graminearum*, except for *C. rosea* CBS 102.94, *P. indica* DSM11827, and *P. williamsii* DAR29830. Due to their slow growth rate, they were cultured for two days at 25 °C then at day 3, a disc of *F. graminearum* was cultured. The growth rate reduction was calculated in the same manner as in the direct plating assay. For each isolate, five replicates were conducted and the whole assay was repeated three times. The effect on pathogen pigments was recorded.

#### 5.3.3. In planta (Pot Experiments) Assay

An in planta assay under controlled greenhouse conditions was developed to evaluate the antagonistic activity of the selected isolates against the GER pathogen. *F. graminearum* PH1 (25 discs of 5 mm diameter each from one week old PDA plate) was cultured at 22 °C for 10 days (12 h light/12 h darkness) on sterile rice which was autoclaved at 121 °C for 15 min in a 500 mL Erlenmeyer flask. The water activity was increased to allow suitable fugal growth by adding 50 mL distilled water for each 125 g rice. Parallel to that, each BCA was cultured in a 300 mL PDB in a 500 mL Erlenmeyer flask covered externally with aluminum folium at 25 °C for two weeks. After incubation, 4 mL were transferred into a sterile 50 mL falcon tube, and stored at 4 °C for coating the seeds (soaking for 2–3 min) prior to seeding into soil. The remaining culture medium was filtered using normal filter paper, and mycelium (12.5 gm ± 1) was re-suspended in 500 mL PBS, and then homogenized using the IKA^®^ homogenizer (yellow line DI 25 basic, IMLAB bvba, Boutersem, Belgium) at low speed. The PBS-containing mycelium was thoroughly mixed with the soil (1:100) in a plastic tray, and covered for 5–7 days to allow sporulation. Before sowing, maize seeds were coated with BCA mycelium suspension, and *F. graminearum* PH1 grown on rice was added to the soil (12.5 g for each 1.25 kg soil or 1:100). The soil was composed of a mixture of clay and sand (3:1, *v*/*v*) added to pure soil (50/50, *v*/*v*), and semi-sterilized at 75 °C in an oven for 4–5 days. Each pot contained 250 g of soil. For each *F. graminearum*/antagonist combination, five replicates were set up (four seeds per pot) in addition to the negative control (only soil) and the positive control (*F. graminearum* PH1). Plants were grown for two weeks at 22 °C, 12 h light/12 h darkness and watered every 2–3 days. After 14 days, plants were harvested, and evaluated by measuring the stem length and weighing the root of each plant from each pot separately. The whole experiment was repeated two times.

### 5.4. Multi-Mycotoxin Quantification Using LC-MS/MS

#### 5.4.1. Mycotoxin Stock Solutions and Other Reagents

All the reagents used during the analysis were of analytical grade. Methanol and acetonitrile (LC-MS grade) were purchased from BioSolve BV (Valkenswaard, The Netherlands), while methanol (HPLC grade) was obtained from VWR International (Zaventem, Belgium). Ammonium acetate, acetic acid (glacial, 100%), hydrochloric acid fuming 37% and formic acid (98–100%) were supplied by Merck (Darmstadt, Germany). Dichloromethane was purchased from Across Organics (Geel, Belgium). Ultrapure water was obtained from a Milli-Q^®^ SP Reagent water system from Millipore Corp (Brussels, Belgium). Individual mycotoxin standards of DON, 15-ADON, DON3G, ZEN, zearalanone (ZAN), and deepoxy-deoxynivalenol (DOM) were acquired from Sigma Aldrich NV/SA (Bornem, Belgium). DOM was used as internal standard for DON, 15-ADON, and DON3G, while ZAN was used for ZEN to compensate the matrix effects for more precise quantification. Stock solutions of DON, ZAN, and ZEN were prepared in methanol while 15-ADON was dissolved in acetonitrile to reach the standard concentration of 1 mg/mL. DOM and DON3G were dissolved in acetonitrile to obtain a standard concentration of 50 μg/mL. The stock solutions of the mycotoxins and internal standards were diluted in methanol except for 15-ADON, DOM, and DON3G, which were diluted in acetonitrile to obtain a working solution with a concentration of 1 ng/μL. The stock standard solutions were stored in a freezer at −20 °C, while working solutions were stored in a fridge at 4 °C. Matrix-matched calibration curves (MMC) of five points (0, 100, 500, 2500, and 5000 µg/kg) were used in order to quantify mycotoxins.

#### 5.4.2. Extraction of Fungal Metabolites

##### Extraction from Culturing Solid Media

Sample extraction of the PDA plates from the contact and volatile assay was done as described by Delmulle et al. (2006) with minor modifications [42]. Briefly, each sample was collected in a 50 mL falcon tube (Sarstedt, Nümbrecht, Germany), a suitable amount of liquid nitrogen was added for sample crushing and homogenization and 2 g were transferred into another falcon tube. The samples were spiked with DOM and ZAN and left for 3 h in a dark place with opened caps to allow solvent evaporation. Five mL of ethyl acetate were added, and samples were subjected to shaking for 15 min using an overhead shaker (Edmund Bühler GmbH, Hechingen, Germany). The upper layer was removed and transferred into a new glass test tube, and 5 mL of dichloromethane were added to the sample tube and subjected for 15 min of shaking. The sample content was filtered using a filter paper (VWR International, Leuven, Belgium) into the same glass test tube containing the ethyl acetate, and the filter paper was washed with few drops of each extraction solvent. The filtrate was evaporated under a gentle stream of nitrogen gas (Biotage–TurboVap LV, Uppsala, Sweden) at 40 °C till complete dryness, and re-dissolved in 200 µL of injection solvent (mixture mobile phase A and B (60/40, *v*/*v*)). Before sample transfer into micro-injection vials, the samples were vortexed for 60 s, and centrifuged at 14,000 rpm for 5 min through centrifugation micro tubes (Merck Millipore, Tullagreen, Ireland). Finally, the samples were transferred to microinjection vials, and injected into the LC-MS/MS.

##### Extraction from Two Week Maize Plants

The two-week old maize plants (the whole plant including the seeds) were collected, the roots were cleaned and washed by dipping in water for 5 s, and dried at 40 °C in the oven for 24 h. The samples were finely crushed using liquid nitrogen, and 8 mL of the extraction solvent (ethyl acetate: formic acid: *v*/*v*) were added to 1 g of each sample (four plants per one pot). The mixture was vortexed for 60 s, shaken for one hour using the overhead shaker, centrifuged at 3500 rpm for 5 min, and 80 µL of the supernatants were diluted with 20 µL of internal standards mixture (10 ng/µL). The final mixture was evaporated under a gentle stream of nitrogen gas at 40 °C till complete dryness, re-dissolved in 200 µL of injection solvent, centrifuged at 10,000 rpm for 5 min, and transferred into injection vials for subsequent LC-MS/MS analysis.

#### 5.4.3. LC-MS/MS Methodology

For mycotoxin detection and quantification, LC-MS/MS analysis was performed using a Waters Acquity UPLC-Quattro Premier XE mass spectrometer (Waters, Milford, MA, USA) in positive electrospray ionization (ESI+) mode. Chromatographic separation was achieved using a Symmetry C18 (150 mm × 2.1 mm, i.d. 5 μm) column with a guard column (10 mm × 2.1 mm i.d.) of the same material (Waters, Zellik, Belgium). Both column and guard were kept at RT. Mobile phases consisting of water/methanol/acetic acid (94/5/1, *v*/*v*/*v*) and 5 mM ammonium acetate (mobile phase A), and methanol/water/acetic acid (97/2/1, *v*/*v*/*v*) and 5 mM ammonium acetate (mobile phase B) were used at a flow rate of 0.3 mL/min with a gradient elution program. This gradient program was as follow; 0–7 min, 95–35% A; 7–11 min, 35–25% A; 11–13 min, 25–0% A; 13–14 min, 0% A; 14–16 min, 0–40% A; 16–26 min, 40–60% A; 26–28 min, 60–95% A. The column was reconditioned for 5 min prior to the next injection. The total analytical run time was 28 min with a pressure that varied between 0 and 5000 psi. The sample injection volume was set at 10 µL. During the LC-MS/MS analysis, the injection solvent was inserted between the blank and spiked samples to avoid any carry-over. Data acquisition and processing utilities included the use of the MassLynx and QuanLynx version^®^ 4.1 software (Micromass, Manchester, UK). To achieve the optimal sensitivity and selectivity of the MS conditions, data acquisition was performed by applying selected reaction monitoring (SRM). For each target analyte, two precursors to product ion transitions were selected. In accordance with Commission Decision (EC) No. 657/2002, laying down the performance criteria of analytical methods, the use of two transitions allowed determination of the ratio between these two transitions (relative ion intensity). This ratio, together with the relative retention time and signal-to-noise ratio, allows the confirmation of the identity of the detected compound. More information on the transition of the different mycotoxins is described in detail by Ediage et al. (2011) [43]. The capillary voltage was 3.2 kV and nitrogen was used as the desolvation gas. Source and desolvation temperatures were set at 150 °C and 350 °C, respectively.

### 5.5. Statistical Analysis

For statistical evaluation, the R software package (R core Team, 2017, Vienna, Austria) version 3.4.2 was used. Firstly, the normality and homoscedasticity assumptions of parametric tests were assessed. As these were not met, a non-parametric Kruskal–Wallis test was used to verify if there were significant differences among groups of data (differences between strains, including the control). In case there was a significant difference (*p*-value < 0.05), a pairwise comparison of the groups using a Dunn test was performed. The data are represented as box plots, which provide a graphical view of the median (horizontal line) and quartiles (Q1 − Q3, box). The upper whisker is located at the smaller of the maximum *x* value and Q3 + 1.5× interquartile range, whereas the lower whisker is located at the larger of the smallest *x* value and Q1 − 1.5× interquartile range. An outlier is defined as a data point that is located outside the whiskers of the boxplot, outside 1.5 times the interquartile range above the upper quartile and below the lower quartile.

## Figures and Tables

**Table 1:** List of the isolated strains from maize stubble and soil in Flanders, Belgium.

	Strain Name	Abbreviation	Source	Accession Number
1	*Clonostachys rosea* (UG 1116, Belgium)	*C. rosea* UG 1116	Maize stubble	KX099625.1
2	*Epicoccum nigrum* (UG 11701, Belgium)	*E. nigrum* UG 1701	Soil	MH277348.1
3	*Epicoccum nigrum* (UG 11702, Belgium)	*E. nigrum* UG 1702	Soil	MH258972.1
4	*Epicoccum nigrum* (UG 11703, Belgium)	*E. nigrum* UG 1703	Soil	MH245107.1
5	*Sordaria fimicola* (UG 1016, Belgium)	S. *fimicola* UG 1016	Soil	KY930619.1
